# A non-equivalent group pilot trial of a school-based physical activity and fitness intervention for 10–11 year old english children: born to move

**DOI:** 10.1186/s12889-016-3550-7

**Published:** 2016-08-24

**Authors:** Stuart J. Fairclough, Bronagh McGrane, George Sanders, Sarah Taylor, Michael Owen, Whitney Curry

**Affiliations:** 1 Physical Activity and Health Research Group, Sport and Physical Activity Department, Edge Hill University, St Helens Road, Ormskirk, Lancs L39 4QP UK; 2 Department of Physical Education and Sports Science, University of Limerick, Limerick, Ireland

**Keywords:** Physical education, Fitness, Physical activity, Enjoyment, Pilot, Perceived competence, Intervention, Schools, Children

## Abstract

**Background:**

PE lessons are the formal opportunity in schools for promotion of physical activity and fitness. This study aimed to evaluate the effectiveness of a pilot PE intervention on physical activity, fitness, and psychosocial outcomes.

**Methods:**

Participants were 139 children aged 10–11 years from four schools. For six weeks children in two schools received a twice-weekly pilot ‘Born to Move’ (BTM) physical activity (PA) and fitness intervention alongside one regular PE lesson. Children in the two comparison (COM) schools received their regular twice weekly PE lessons. Outcomes were lesson time and whole-day light (LPA), moderate (MPA), vigorous (VPA), and MVPA, and sedentary time, muscular fitness, cardiorespiratory fitness (CRF), and lesson-specific perceived exertion, enjoyment, and perceived competence. Outcomes were assessed at baseline (T0), midway through the intervention (T1), and at the end (T2) using ANOVAs and ANCOVAs. Intervention fidelity was measured using child and teacher surveys at T2 and analysed using Chi-square tests.

**Results:**

The BTM group engaged in moderate PA for significantly more lesson time (29.4 %) than the COM group (25.8 %; *p* = .009, d = .53). The amount of moderate-to-vigorous PA (MVPA) during the T1 BTM lesson contributed 14.0 % to total MVPA, which was significantly more than the COM group’s T1 PE lesson (11.4 %; *p* < .001, d = .47). The BTM group were significantly more active during the whole-day (*p* < .05) and the school-day (*p* < .01). In both groups push-up test performance increased (*p* < .001) and CRF test performance decreased (*p* < .01). Perceived exertion, enjoyment, and perceived competence increased in both groups (*p* < .05), but the BTM group rated their enjoyment of the T1 BTM lesson higher than the COM group rated their PE lesson (*p* = .02, d = .56). The children’s and teachers’ responses to the intervention indicated that the delivery aims of enjoyment, engagement, inclusivity, and challenge were satisfied.

**Conclusions:**

The BTM pilot programme has potential to positively impact on physical activity, fitness, and psychosocial outcomes. Further, BTM was enjoyed by the children, and valued by the teachers. This study can inform the design of a modified larger-scale cluster RCT evaluation.

## Background

Physical activity in childhood conveys many health benefits across the physical, psychological, social, and emotional domains [1]. Despite evidence highlighting the positive health effects of active lifestyles [2–5], the majority of children and young people do insufficient physical activity of at least a moderate intensity (MPA) to achieve current guidelines for health [1]. Moreover, physical inactivity and increased prevalence of sedentary behaviours in youth are associated with negative health indicators such as obesity and type 2 diabetes [6]. Moderate-to-vigorous physical activity (MVPA) positively influences cardiorespiratory and muscular fitness, which promote a number of health-related benefits [2, 7], but both of which have declining levels among youth [8, 9]. Further, accumulation of light intensity physical activity (LPA) in place of sedentary time may benefit children’s health through associations with adiposity and cardiometabolic risk [10–12]. Thus, physical activity intervention efforts in youth are warranted, particularly when they promote MVPA and fitness. Intervention approaches set in and delivered through school environments hold promise [13], as they can facilitate a range of physical activity and fitness opportunities, including discretionary periods between lessons and at break times, and through more structured and formal periods such as physical education (PE) lessons. Evidence indicates that school-based interventions can be effective in enhancing physical activity, cardiorespiratory and muscular fitness, psychosocial outcomes associated with physical activity such as enjoyment, and other markers of health status in youth [2, 13–16].

PE lessons are the formal opportunity in schools for direct delivery of health-related physical activity and fitness. For this reason PE is often viewed as the primary vehicle for promoting these outcomes in schools [17]. A recent systematic review and meta-analysis concluded that PE-based interventions result in children spending 10.4 % more lesson time in moderate-to-vigorous physical activity (MVPA) compared to regular PE lessons, which could have a significant contribution to total daily physical activity levels [18]. Moreover, there is evidence that PE interventions can positively impact on health-related fitness [19, 20] and motivational constructs, such as enjoyment [21]. In England, PE is a mandatory subject in schools through all years of compulsory schooling (ages 5 through 16 years). Schools align their curricula to the National Curriculum programmes of study [22], which typically emphasise traditional competitive games-based activities. This narrow curriculum structure is however, not suited to all children, some of whom prefer activities that are more movement and exercise oriented. Such activities that explicitly promote physical activity, fitness, and health may appeal to, and also reflect recreational participation of a wider range of youth than more traditional PE activities [23].

This study aimed to evaluate the effectiveness of a pilot PE intervention programme on selected physical activity, fitness, and psychosocial outcomes known to influence physical activity engagement. The specific objectives were to evaluate the effectiveness of the pilot intervention on children’s: objectively measured LPA, MPA, vigorous physical activity (VPA), MVPA, and sedentary time during lessons and during the whole-day, muscular fitness, cardiorespiratory fitness, lesson enjoyment, perceived exertion, and perceived competence. We also sought to examine intervention fidelity, and to provide the necessary information to calculate the sample size for a cluster RCT evaluation of the intervention programme. The study is reported in accordance with the Transparent Reporting of Evaluations with Nonrandomized Designs (TREND) statement [24].

## Methods

### Design and recruitment

The study was a non-equivalent groups pilot trial of a PE-based physical activity and fitness intervention in primary schools and was delivered between November and December 2015. Homogenous purposive sampling was used to recruit schools local to the university that were known to advocate PE and physical activity initiatives promoting children’s health and wellbeing. Four co-educational primary schools from West Lancashire in north-west England were initially identified from 51 schools in local School Sport Partnership. Following meetings between the School Sport Partnership Manager, the principal investigator, and school head teachers all four of the schools agreed to participate in the project. The schools were located in areas of relatively low deprivation according to their location postal codes (i.e., deciles 7–9 of the 2015 English Indices of Multiple Deprivation) [25]. The percentage of children eligible for free-school meals ranged from 5.5 to 23.1 %, which was lower than the national average of 26.6 %.

All Year 6 children (age 10–11 years; *n* = 147) in the schools were informed about the project by their class teachers and those children that expressed an interest were given an information pack to take home and share with their parents/carers. Written informed consent and assent were required from the parents/carers and children respectively, before children could participate in the project. These documents were returned to the research team via the schools in accordance with the project approvals granted by the University Research Ethics Committee (reference # SPA-REC-2015-182). Children were included if they provided the required informed consent, assent, and medical screening forms which indicated an absence of any medical conditions or disabilities which prevented participation in the data collection and/or regular PE lessons.

#### Allocation to conditions

Two of the four schools were able to reorganise their timetables to accommodate the intervention lessons, but due to time pressures to deliver numeracy and literacy, and demands on the limited indoor spaces for PE, this was not possible in the other two schools. This meant that the intervention schools were allocated prior to baseline data being collected to allow time for reorganisation of curricula and class allocations to indoor PE spaces. The two schools that did not receive the intervention acted as comparisons delivering their regular curricula. All four schools were situated in similar geographical areas so the children were as closely matched by catchment areas and exposure to community-based initiatives promoting physical activity, fitness, health and wellbeing.

**Fig. 1:**
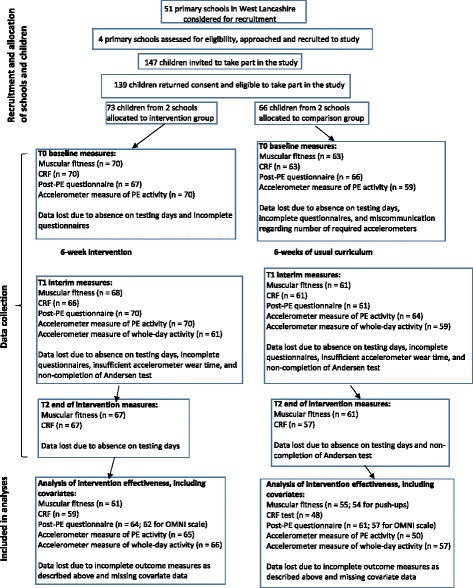
Flow of participants through the study

Informed consent to take part was obtained from 139 children (94.6 % participation rate; 73 children in the intervention group, 66 in the comparison group; Fig. 1). Non-consenting children participated in BTM and PE lessons with the rest of their classes but no research data was collected from them. Sample size was not determined by a formal calculation as this was deemed unnecessary for a pilot study of this nature [26, 27]. The sample was representative of the target study population (i.e., Year 6 children in West Lancashire who regularly participate in PE lesson) and was based on relatively equal class sizes in the participating schools. Moreover, our sample size was larger than the median of 30 participants per arm reported for pilot studies in the UK with continuous variable endpoints [26].

### Intervention

This study piloted a structured class-based physical activity and fitness programme entitled Born to Move (BTM; http://www.lesmills.com/borntomove). BTM is an age-adapted portfolio of class-based physical activity and fitness programmes set to music with an emphasis on enjoyable and inclusive activity. The classes teach age-appropriate motor skills designed to improve health-related and skill-related fitness. The activities were delivered through eight combined movement categories (Table 1) set to contemporary music tracks of varying tempo. The format of the lessons was specifically designed for mixed-sex groups of children in the 8–12 years age range. The lessons were designed to be enjoyable, differentiated, and inclusive, yet challenging and requiring concentration and physical effort. The lessons were intended to last for at least 30 min and were taught in the schools’ PE halls to the intact mixed-sex and mixed ability Year 6 classes. All of the BTM lessons were taught free of charge by one trained female instructor of 10 years experience who is a BTM UK instructor trainer. She adhered closely to the child-centred principles that are central to the BTM instructor training. These include understanding how children learn, group observation and interaction, role modelling, demonstrating a positive attitude to physical activity, and having flexible teaching strategies to meet the needs of different groups and individuals. The intervention’s emphasis on enjoyable and inclusive lesson content delivered by an expert instructor is consistent with the Youth Physical Activity Promotion Model (YPAPM [28]) which provided a conceptual framework for the intervention evaluation. The YPAPM recognises that children’s physical activity is influenced to varying degrees by predisposing, enabling, reinforcing, and personal demographic factors, and their mutual interactions. Predisposing factors in particular are highlighted in the model as significant predictors of physical activity participation [29], and include self-evaluative constructs (e.g., perceived competence) and a cognitive assessment of the perceived outcomes of activity (e.g., enjoyment, interest) [28].

**Table 1:** Born to Move intervention movement categories

Movement	Description
Move	Simple movements for warm-ups and active rests
Punch	Combinations of punches, strikes, and high knees designed to raise heart rates
Kick	Technical kicks taught with an emphasis on accuracy and focus
Jump	Plyometric and sport-based movements to increase muscular fitness
Dance	Dance sequences which gradually progress in complexity
Core	Fun-based full body movements using body weight exercises
Games	Interactive and fun games designed to fully engage the children
Yoga	Simple yoga sequences designed to promote flexibility and concentration

Each week for six consecutive weeks, children in the intervention schools received two BTM lessons in addition to one regular PE lesson. The regular PE lessons lasted between 30 and 45 min and were taught by the usual Year 6 class teachers or teaching assistants in accordance with the planned PE curricula for those weeks. The BTM instructor and Year 6 teachers in the BTM schools were fully aware that their classes were receiving the intervention. The research team were not involved in implementing any aspect of the intervention and knew the children only by their study identifier numbers. Children from the two comparison schools (COM) did not receive the BTM intervention and instead took part in their regular twice-weekly PE lessons which covered activities including netball, benchball, hand-tennis, cricket, dance, and fitness circuits. These lessons were taught by the usual Year 6 class teachers and were intended to last between 30 and 45 min. At the end of the six-week intervention all consenting children in the BTM and COM groups received a £10 gift voucher that could be used in a variety of online and high street retailers. Following completion of the project BTM after-school clubs were planned in all four participating schools for the 2016 summer term, after which a BTM community programme would be implemented in the local leisure centre. These programmes would be delivered by trained instructors based in the schools and local community.

Baseline data collection (T0) commenced in the week of 19th October 2015 which was followed by a one-week half-term break. The intervention pilot began in the week of 2nd November 2015 and ended the week of 7th December 2015. Data were also collected halfway through the intervention (T1) and at the end (T2).

### Outcome measures

Outcome measures to evaluate the effectiveness of the pilot BTM programme were assessed at T0 before the 6-week intervention, at T1, and where measured, at T2. The primary study outcomes were: LPA, MPA, VPA, MVPA, and sedentary time during lessons, muscular and cardiorespiratory fitness, and enjoyment, perceived exertion, and perceived competence during lessons. Secondary outcomes were: whole-day, school-day, and after-school through evening physical activity and sedentary time. These outcomes were measured on a selected BTM or PE lessons day rather than averaged across the entire week. All data collection measures were administered by trained researchers who were un-blinded to the classes’ allocations to the BTM and COM groups.

#### Socioeconomic status

Area level socio-economic status was calculated using the 2015 Index of Multiple Deprivation (IMD) [25] derived from parent/carer reported home postal codes. Higher IMD scores represent areas of higher relative deprivation and lower scores indicate areas of lower relative deprivation.

#### Anthropometrics

All children undertook anthropometric assessments at the school sites according to standard procedures [30]. Height and sitting height were measured to the nearest 0.1 cm using a portable stadiometer (Seca 213 height measure, Seca UK, Birmingham, UK). Body mass was measured to the nearest 0.1 kg using calibrated digital scales (Seca 877 digital scales, Seca UK, Birmingham, UK). Body mass index (BMI) was calculated from height and body mass as a proxy measure of body composition (kg · m^2^), and BMI z-scores were assigned to each child [31]. Age and sex-specific BMI cutpoints were used to classify children’s weight status [32]. Gender-specific regression equations [33] were used to predict children’s maturity offset (i.e., age from peak height velocity), which was used as a proxy measure of somatic maturation.

#### Muscular fitness

The FITNESSGRAM push-up test was administered as a measure of upper body muscular fitness [34]*.* The children completed as many repetitions as possible and the test was terminated if the children did not maintain the prescribed cadence or they did not achieve a 90° angle with the elbow. The standing long jump test was used as a measure of lower body muscular fitness [35]*.* The longest distance jumped was recorded in cm from the best of three attempts.

#### Cardiorespiratory fitness: Andersen test

Children completed a modified version of the Andersen Test which is a 10-min 20 m intermittent shuttle run/rest test of cardiorespiratory fitness (CRF; [36]). The test has recently been shown to be valid and reliable for providing group level estimates of CRF in 10 year old children [37]. Due to indoor space restrictions in the schools, the distance between the two lines was modified to 10 m rather than the usual 20 m. Prior advice on this modification was taken from the test’s author, who highlighted that reducing the shuttle run distance would increase the number of times the children would need to turn, which would likely lead to an underestimation of CRF because of the additional energy cost associated with the extra turning (personal correspondence, October 2015). As all of the schools would use the 10 m adaptation of the test and therefore between- and within-group differences would be consistent, it was decided to proceed with this modification. The test consisted of the children shuttle running from one end line to the other for 10 min. Every 15 s a digital audio cue sounded which signaled for the children to stop and rest, or resume running. The research team counted the number of shuttles completed in 10 min, after which the test ended. The children were encouraged to keep running at their own pace as per the test protocol [37]. Total distance completed was recorded and peak VO_2_ was estimated using sex-specific equations [37].

#### Post-lesson questionnaire measures

At the end of BTM and PE lessons children were asked to rate their perceived exertion for the lesson overall by completing the Children’s OMNI Perceived Exertion Scale for Stepping Exercise [38]. Enjoyment and perceived competence in relation to BTM or PE lessons were assessed using four items from the short-form Intrinsic Motivation Inventory (IMI) [39], which measures underlying constructs of intrinsic motivation and has been used previously in PE research [40, 41]. The short-form IMI has demonstrated satisfactory construct validity [39] and was developed for use in school settings when data collection time is limited. Items were modified to make them situational-specific [42] and responses were recorded on a 7-point Likert scale.

### Objectively measured physical activity and sedentary time

Physical activity and sedentary time were objectively measured using triaxial accelerometers (Actigraph GT9X, ActiGraph LLC, FL, USA). The Actigraph monitor has demonstrated validity and reliability in estimating children’s physical activity and sedentary time across a range of ages [43]. Children wore the monitors on their non-dominant wrists during selected T0 and T1 PE and/or BTM lessons. On a selected T1 day when children were scheduled for BTM or PE, the monitors were also worn from waking to bedtime to investigate the contribution of the BTM and PE lessons to overall physical activity and sedentary time. It was not logistically possible to measure whole-day activity during T0 because timetabling of PE between schools occurred on the same day or on consecutive days. As a consequence, the limited number of available accelerometers meant that concurrent monitoring in different schools was not possible. Greater timetable flexibility in the second half of the term allowed whole-day monitoring during T1. To be included in the whole-day analysis, children needed to wear the accelerometer for at least 540 min. Accelerations were recorded at a frequency of 30 Hz and were subsequently converted to counts per 1 s epochs. Percentages of BTM/PE lesson time, and whole-day, school-day, and after-school through evening minutes spent sedentary, and in light (LPA), moderate (MPA), vigorous (VPA), moderate-to-vigorous physical activity (MVPA), and total physical activity (total PA; i.e., LPA through to VPA) were calculated based on wrist count cutpoints developed by Chandler et al. [44]. Data reduction and preliminary analysis of the accelerometer data were conducted in Actilife (version 6.11.5, theActigraph.com, Pensacola, FL).

### Intervention fidelity

To assess intervention fidelity the number and duration of planned BTM lessons were recorded and at T2 the children in the BTM schools completed a 14-item survey which asked about perceptions of the BTM lessons, how they were taught, and what impact they had had. A simple ‘yes/no’ structure was used with children asked about their perceptions of BTM. Questions related to areas such as perceived challenge (e.g., “Did you find the BORN TO MOVE lessons a challenge?”), motivation (e.g., “Did the BORN TO MOVE teacher motivate you to try hard?”, and “Did doing the BORN TO MOVE lessons to music motivate you to try hard?”), and adaptability (e.g., “Did the BORN TO MOVE teacher change or adapt any movements or skills that seemed difficult so they were easier to do?”). The intervention class teachers were asked to complete a free-text evaluation of the programme, which asked them to comment on the delivery of the BTM lessons, and the intervention’s influence on the children’s lesson engagement and competence, classroom learning, and general wellbeing.

### Data analysis

#### Preliminary analysis

Preliminary analyses checked the distribution of the variables by group (BTM vs. COM). Across the three time points, Kolmogorov-Smirnov tests revealed that the majority of variables were not normally distributed. Logarithmic and reciprocal transformations were applied to the data and while these normalised some of the variables for at least one time point, this outcome was not consistent across all variables and time points. Thus, on the basis that ANOVA models are generally robust to violations of the normality assumption [45] it was decided to proceed with parametric tests. The exception to this was where the data were categorical, or where the data were skewed and there was no need to include covariates in the main analyses.

#### Baseline data analysis

The initial analysis of T0 data investigated equivalence between the BTM and COM groups. Between-group differences in the primary and secondary outcomes were analysed using Mann–Whitney tests or independent t-tests, depending on the distribution of the outcome data. Study outcomes did not differ at T0 between the BTM and COM groups (*p* > .05) with the exception of IMD scores (BTM > COM; *p* = .004), and PE/BTM lesson accelerometer data for physical activity (LPA, MPA, VPA, MVPA, and total PA; BTM > COM; *p* < .001), and sedentary time (BTM < COM; *p* < .001). Subsequent analyses of PE/BTM lesson physical activity and sedentary time included baseline physical activity or sedentary time values as covariates. Group (BTM vs. COM) x sex ANOVAs were employed to check for differences in outcomes between boys and girls in the BTM and COM groups. For push-ups there was a significant sex x group interaction effect (*p* = .021) which indicated that the BTM boys’ performance was superior to the girls, whereby the COM boys and girls performed similarly. The same sex x group interaction (*p* = .001) and data trends were observed for standing long-jump. As a consequence, sex was included as a covariate in the main analyses of push-ups and standing long jump.

#### Main analysis

As children were nested within schools, the variability between school-level data was examined via multilevel analyses. Little variability was seen between schools for all outcome measures (all ICCs < .05 [46]), therefore further multilevel analyses were deemed unnecessary, and child-level data were analysed to evaluate intervention effectiveness. Intervention effects on the primary and secondary outcomes were assessed using group (BTM, COM) x time (T0, T1, T2) ANOVAs or ANCOVAs. Appropriate prognostic covariates were included in these analyses based on their known influence on the outcomes [47]. Analysis of push-up performance was adjusted for sex, BMI z-score [48, 49] and IMD score [50, 51], while sex, body mass [49], leg length [35, 52], and IMD score [50, 51] were covariates in the analysis of standing long-jump performance. The effect of the intervention on CRF was assessed through analysis of total distance covered during the Andersen test, and estimated peak VO_2_. The analysis of shuttle run distance was adjusted for BMI z-score [8] and IMD score [50, 51]. Estimated peak VO_2_ expressed as ml⋅kg⋅min^−1^ was adjusted for IMD score. The post-BTM and PE lesson questionnaire scores were analysed using group x time ANOVAs with the exception of the analysis of perceived exertion which used ANCOVA with adjustment for BMI z-scores. One-way between-group ANCOVAs compared differences in the absolute and percentage of BTM or PE lesson time spent in different physical activity intensities. These analyses controlled for T0 values of the outcomes, and BMI z-scores. The percentage contribution of the BTM and PE lessons to overall MVPA and to the 60 min/day MVPA minimum recommendation for health [1] were compared using Mann–Whitney tests. Between-group differences in time spent in objectively measured whole-day, school-day, and after-school through evening physical activity and sedentary time were assessed using one-way ANCOVAs with adjustment for IMD scores, BMI z-scores, and accelerometer wear time.

#### Sample size analysis

MPA during PE was chosen as the main outcome for sample size estimation because the BTM programme generally involves moderate intensity activities more so than moderate-to-vigorous intensity activities. Potential sample sizes for a future RCT were estimated using PINT 2.12 statistical power analysis software [53]. Calculations were based on several key assumptions: 1) a future RCT would have one intervention and one control arm, 2) a common standard deviation, and 3) the smallest effect size worth identifying for between-group difference would be 6.5 min during a PE lesson. Based on our observed MPA and VPA pilot data this increase would amount to 50 % of BTM lesson time in MVPA which reflects recommendations for health-enhancing PE [54, 55].

#### Sub-group analyses

Sub-group analyses on the primary and secondary outcomes were conducted to evaluate whether the effects of the BTM pilot differed between boys and girls. These analyses involved sex-specific analyses of the primary outcomes over time with adjustment where appropriate for the same covariates as in the main analyses.

#### Intervention fidelity

Percentage responses and Chi-Square tests were used to evaluate the children’s responses to the end of programme survey. Teachers’ free-text responses were grouped according to the main themes of lesson enjoyment, teacher delivery, child engagement, and general wellbeing.

All quantitative data were entered into a Microsoft Excel database (Excel for Mac version 15.17, Microsoft, Redmond, WA). Following data cleaning and checking the data were uploaded to IBM SPSS Statistics (version 22, IBM Corp., Armonk, NY) for analysis. Alpha was set at *p* < .05, and where effect sizes (d) were calculated their magnitude was described according to Cohen [56].

## Results

### Descriptive characteristics of the participants

BTM and COM children were well matched across the majority of measures (Table 2). The children typically resided in areas of relatively low deprivation though there was some variation in this with 19 % of children living in the lowest five IMD deciles nationally. The overweight and obesity prevalence of the children participating in this project (BTM = 25.7 % and COM = 20.6 %) was lower than the local norm in West Lancashire of 35 %.

**Table 2:** Descriptive characteristics and T0 lesson duration, physical activity and sedentary time for the BTM and COM groups (median and inter-quartile range unless stated otherwise)

Descriptive measure	BTM		COM	
	n	Median (IQR)	n	Median (IQR)
Age (y)	73	10.7 (0.6)	66	10.7 (0.6)
Height (cm)	70	142.0 (8.3)	63	143.6 (7.4)
Weight (kg)	70	36.6 (13.7)	63	37.6 (8.3)
BMI (kg/m^2^)	70	17.4 (5.2)	63	18.2 (3.6)
BMI z-score	70	0.30 (0.88)	63	0.50 (1.50)
Weight status: Normal weight (%)	70	74.3	63	79.4
Weight status: Overweight/obese (%)	70	25.7	63	20.6
Maturity offset (y)	70	−2.6 (1.8)	63	−1.8 (2.0)
IMD score	72	11.0 (7.0) ***	66	6.0 (6.5)
PE lesson duration (min)	73	50.0 (17.0) ***	66	28.0 (6.0)
LPA (% PE lesson time)	70	28.5 (7.4) ***	59	24.2 (8.1)
MPA (% PE lesson time)	70	32.7 (4.5) ***	59	23.4 (11.9)
VPA (% PE lesson time)	70	17.0 (9.8) ***	59	8.4 (8.3)
MVPA (% PE lesson time)	70	51.3 (9.3) ***	59	32.0 (17.6)
Total PA (% PE lesson time)	70	81.3 (7.0) ***	59	57.4 (24.3)
Sedentary time (% PE lesson time)	70	18.7 (6.9) ***	59	42.6 (24.8)

*** *p* < .001

### Primary outcomes

#### Physical activity and sedentary time during BTM and PE lessons

The mean BTM lesson and PE lesson durations were 43.6 ± 2.2 min and 36.1 ± 4.7 min, respectively. The BTM group were sedentary for 6.6 % less lesson time than the COM group (*p* = .055, d = .40; Table 3). Furthermore, the BTM group engaged in MPA for significantly more lesson time (3.6 %; *p* = .009, d = .53) than the COM group. There were no significant differences in the percentage of lesson time spent LPA, VPA, MVPA, or total PA. The BTM group engaged in significantly more minutes of LPA, MPA, MVPA, and total PA than COM group peers (*p* < .001; Table 3). The amount of MVPA that the children engaged in during the T1 BTM lesson contributed 14.0 % to their total MVPA for that day, which was significantly more than the contribution of the T1 PE lesson to the COM group’s whole-day MVPA (11.4 %; *p* < .001, d = .47). Furthermore, MVPA during the T1 BTM lesson represented 31.8 % of the daily minimum of 60 min MVPA guideline [1], which was significantly more than the 25.6 % contribution from the T1 PE lesson (*p* < .001, d = .60).

**Table 3:** T1 adjusted† PE and BTM lesson physical activity and sedentary time outcomes (means and 95 % confidence intervals)

Outcome	BTM	COM		
	(*n* = 65)	(*n* = 50)	p	d
LPA (% lesson time)	26.2	25.2	.25	.36
	(25.1, 27.30	(24.0, 26.4)		
MPA (% lesson time)	29.4	25.8	.009	.53
	(27.8, 31.0)	(23.9, 27.6)		
VPA (% lesson time)	14.1	16.1	.27	.20
	(12.0, 16.3)	(13.6, 18.6)		
MVPA (% lesson time)	44.7	40.4	.15	.15
	(41.4, 47.90	(36.5, 44.3)		
Total PA (% lesson time)	71.4	64.8	.055	.32
	(67.7, 75.2)	(60.4, 69.3)		
Sedentary (% lesson time)	28.6	35.2	.055	.40
	(24.9, 32.3)	(30.7, 39.6)		
LPA (min × lesson^−1^)	11.8	8.6	.001	1.28
	(11.1, 12.5)	(7.8, 9.5)		
MPA (min × lesson^−1^)	13.6	8.3	.001	1.48
	(12.7, 14.4)	(7.3, 9.3)		
VPA (min × lesson^−1^)	5.7	6.2	.54	.06
	(4.9, 6.6)	(5.2, 7.3)		
MVPA (min × lesson^−1^)	19.6	14.1	.001	.78
	(18.1, 21.1)	(12.2, 16.0)		
Total PA (min × lesson^−1^)	33.0	20.7	.001	1.17
	(31.0, 34.9)	(18.3, 23.1)		
Sedentary (min × lesson^−1^)	12.9	12.0	.38	.13
	(11.7, 14.0)	(10.7, 13.4)		

†Adjusted for T0 outcome values and BMI z-scores

#### Muscular fitness

Push-up test performance improved overall regardless of group (*p* < .001, d = .81; Table 4). The BTM group improved by 111 % between T0 and T2 compared to the COM group’s 68 %. Although a significant time x group interaction effect was observed (*p* = 0.02), simple effects analyses revealed no significant between-group differences at each time point. Modest non-significant improvements in standing long jump performance were observed in the BTM group (4.2 % increase) relative to the COM group (0.8 % increase).

**Table 4:** Adjusted fitness and questionnaire outcomes (means and 95 % confidence intervals)

Outcome	BTM Group				COM Group				
	n	T0	T1	T2	n	T0	T1	T2	Effects
Push-ups	61	5.7	9.6	12.1	54	6.4	12.3	10.7	Time, *p* < .001, d = .81
		(4.4, 7.1)	(7.6, 11.6)	(9.8, 14.4)		(4.9, 7.9)	(10.2, 14.4)	(8.2, 13.1)	
Standing long jump (cm)	61	139.0	142.1	144.8	55	144.2	145.5	146.3	NS
		(133.8, 144.1)	(136.2, 147.9)	(138.9, 150.7)		(139.8, 150.7)	(139.3, 151.80)	(140.1, 152.6)	
Andersen test distance (m)	59	804.7	789.3	753.5	48	809.5	800.8	779.0	Time, *p* = .004, d = .30
		(782.4, 827.0)	(768.1, 810.5)	(731.4, 775.7)		(784.6, 834.5)	(777.1, 824.5)	(754.2, 803.8)	
Estimated peak VO_2_ (ml/kg/min)	59	47.3	46.3	44.4	48	47.8	47.3	46.0	Time, *p* = .001, d = .18
		(45.4, 49.1)	(44.5, 48.2)	(42.5, 46.2)		(45.7, 49.5)	(45.2, 49.1)	(43.9, 48.1)	
Enjoyment	64	5.5	6.1	-	61	5.5	5.7	-	Time, *p* = .001, d = .38; T1 BTM > COM, *p* = .02, d = .56
		(5.1, 5.8)	(5.9, 6.4)			(5.2, 5.9)	(5.4, 6.0)		
Perceived exertion	62	2.0	3.0	-	57	2.3	3.9	-	Time, *p* < .001, d = 1.1
		(1.5, 2.6)	(2.4, 3.6)			(1.7, 2.9)	(3.3, 4.5)		
Perceived competence	64	5.9	6.2	-	61	5.7	5.9	-	Time, *p* = .012, d = .40
		(5.7, 6.2)	(6.0, 6.4)			(5.5, 6.0)	(5.7, 6.1)		

#### Cardiorespiratory fitness

The total distance completed during the Andersen shuttle run test decreased significantly in both groups at each measurement point (*p* = .004, d = .03; Table 4). Resultant estimates of peak VO_2_ also declined in both groups over time (*p* = .001, d = .18). There were no significant group x time interaction effects.

#### Post-lesson questionnaires

Overall, the children rated the T1 lessons as more enjoyable than the T0 lessons (*p* = .001, d = .38). A significant group x time interaction was observed for lesson enjoyment (*p* = .049) which indicated that the BTM group rated their enjoyment of the T1 BTM lesson higher than the COM group rated their PE lesson (*p* = .02, d = .56). Overall time effects but no group x time interactions were evident for perceived exertion (*p* < .001, d = 1.1) and perceived competence (*p* = .012, d = .40).

### Secondary outcomes

#### Whole-day physical activity and sedentary time

**Fig. 2:**
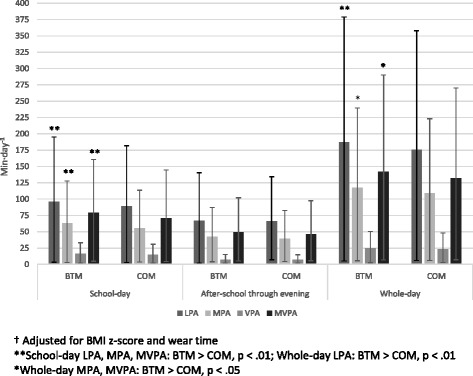
T1 adjusted† school-day, after-school through evening, and whole-day physical activity outcomes (means and 95 % confidence intervals)

On the day when the children wore the accelerometers during waking hours, wear time was 13.1 h and 13.4 h for the BTM and COM groups, respectively. The BTM group accumulated significantly more LPA (11.3 min difference; *p* = .006, d = .21; Fig. 2), MPA (8.3 min difference; *p* = .026, d = .15), MVPA (9.8 min difference; *p* = .044, d = .14), and total PA (15.8 min difference; *p* = .033, d = .18) than the COM group. There were no differences in VPA, but the BTM group spent 21.1 fewer minutes than the COM group in sedentary activity (*p* = .008, d = .39).

#### School day, and after-school through evening physical activity and sedentary time

During the school day the BTM group accumulated significantly more LPA (6.8 min difference; *p* = .001, d = .48; Fig. 2), MPA (7.0 min difference; *p* = .001, d = .48), MVPA (8.3 min difference; *p* = .007, d = .38), total PA (13.1 min difference; *p* = .002, d = .49), and significantly less sedentary time (17 min difference; *p* = .001, d = .59) than the COM group. There were no differences in school day VPA. In contrast, after-school and evening physical activity and sedentary time were similar for both groups (*p* > .05).

### Sample size analysis

For a trial powered to detect a 6.5 min between-group difference in MPA during PE, 16 schools with 23 children per school would be required (*n* = 368; power = .80, α = .05). We calculated the school-level ICC (0.014) for MPA during PE at T2 (95 % CI = < .001 to 0.072) and used the upper 95 % CI in our estimate. A recent review highlighted the mean attrition for child physical activity interventions to be 11.5 % (range 0 – 30 %) [57]. Therefore, a future trial using a conservative 20 % attrition rate would require a sample of *n* = 442 children.

### Sub-group analyses

Exploratory analyses were conducted to investigate whether the intervention effects differed between the BTM boys and girls. Significant sub-group effects are reported. Relative to T0, BTM girls’ push-up test performances improved by 51 % at T1 and by 137 % at T2 (*p* < .001, d = 1.02). An improvement of 57 % was observed between T1 and T2 (*p* = .005, d = .50). Boys’ push-up test performances improved by 78 % between T0 and T1 (*p* < .001, d = .85), and by 93 % between T0 and T2 (*p* < .001; d = 1.03), with little change between T1 and T2. The boys’ BTM group total distance completed during the Andersen shuttle run test decreased significantly at T2 compared to T0 (−41.9 m; *p* < .001, d = .60) and T1 (−27.8 m; *p* = .034, d = .33). Estimates of boys’ peak VO_2_ also declined at T2 relative to T0 (−2.3 ml · kg∙min^−1^; *p* < .001, d = .26) and T1 (−1.5 ml · kg∙min^−1^; *p* = .02, d = .12). Girls (*p* = .028, d = .75) and boys (*p* = .005, d = .70) in the BTM group reported significantly higher enjoyment scores during the T1 BTM lessons compared to the T0 PE lessons. Boys reported higher perceived exertion during the T1 BTM than during the T0 PE lessons (*p* = .003, d = 1.01) but the difference in girls was negligible. Girls’ perceived competence was higher during the T1 BTM lessons relative to the T0 PE lessons (*p* = .015, d = .51) but there were no differences among the boys. During the T0 PE lessons BTM girls engaged in significantly more MPA (*p* = .004, d = .49), VPA (*p* < .001, d = .48), MVPA (*p* < = .001, d = .78), and less sedentary activity (*p* < .001, d = 1.08) than during the T1 BTM lessons. Similarly, in comparison to the T1 BTM lessons, the BTM boys engaged in significantly more LPA (*p* = .006, d = 1.04), MPA (*p* = .002, d = .80), VPA (*p* = .001, d = .43), MVPA (*p* < .001, d = .65), and less sedentary activity (*p* < .001, d = 1.30) during the T0 PE lessons.

### Intervention fidelity

In each BTM school 12 BTM lessons were scheduled over the 6-week programme, with each lesson lasting for a minimum of 30 min to ensure the lesson objectives could be achieved. All 24 lessons were delivered as planned and the mean lesson duration was 43.6 ± 2.2 min which indicates that sufficient time was available to meet the planned objectives. All 73 children assigned to the BTM intervention condition received the intervention, though as attendance registers were not taken during classes, it is unknown how many children received the full dose of 24 lessons.

The BTM lessons included activities focused on health and skill-related fitness, and aimed to be enjoyable, engaging, and inclusive, yet challenging. The children’s responses to the T2 survey provided insight into how well these aims had been had achieved. All the children indicated that they enjoyed the BTM lessons (*p* < .001). All of the girls and 95 % of boys found the lessons interesting (*p* < .001), and around two-thirds of the children thought that the BTM lessons were physically challenging. Between 86 and 100 % of the children thought that after completing the BTM lessons they felt fitter, stronger, and healthier (*p* < .001). All of the children indicated that the BTM teacher made the lessons fun (*p* < .001), and the vast majority (100 % of girls, 92 % of boys) felt that she motivated them to try hard (*p* < .001). Similarly, 90 % of girls and 84 % of boys felt that doing the BTM lessons to music also motivated them to try hard (*p* < .001). Most of the children (59 % of girls, 68 % of boys; *p* < .05) recognized that the teacher changed or adapted the BTM movements or skills when necessary so as to differentiate to the classes’ abilities, and over 94 % of the children felt that they could perform the moves and skills correctly at the end of the 6 weeks (*p* < .001). A large proportion of the children (86 % girls, 79 % boys) felt that they were able to concentrate better on their class work following the BTM lessons (*p* < .001). Finally, 90 % of girls and 87 % of boys stated that they would take part in a BTM after-school club if one was available (*p* < .001).

The class teachers in the BTM schools observed the lessons over the duration of the programme, and mirroring the views of the children, also commented positively about the BTM teacher (“motivational and inspiring”), the music, and how the lessons were fun. The teachers also noted how the BTM teacher and lesson content provided challenge and “really encouraged the children to value their own efforts” and “to push themselves”. The teachers stated how the girls and boys engaged equally well in the lessons, though initially some of the less confident boys were reluctant to stand towards the front of the group. There was a strong belief that the BTM lessons could have a “huge impact on the children academically, socially, and (help to) build their confidence”. Teachers also made reference to seeing the children grow in confidence during and sometimes outside of the BTM lessons. In one school this improvement in confidence was evident among children who previously were disengaged during regular PE classes, but who “have commented how much they have enjoyed the (BTM) sessions”.

## Discussion

This study aimed to evaluate the effectiveness of a pilot school-based physical activity and fitness intervention on physical activity, fitness, and psychosocial outcomes. Significant intervention effects were observed for MPA during lessons, lesson enjoyment, as well as whole-day and school-day physical activity and sedentary time.

### Physical activity and sedentary time during BTM and PE lessons

The BTM group spent more BTM lesson time in MPA than the COM group did during PE. The corresponding absolute difference in MVPA lesson engagement was 4.5 min. This was in line with the ~4.5 min difference between intervention and regular PE MVPA reported in a review of elementary school PE physical activity [58], but was less than the 10.4 % difference reported in a more recent systematic review and meta-analysis of MVPA during PE interventions [18]. In this review over 25 % of the interventions were based on PE delivery through ‘fitness infusion strategies’ [18]. The nature of the studies included under this heading suggests that the intervention lessons involved integration of high-intensity CRF-promoting activities. The BTM intervention employed variable intensity activities and movements such as CRF fitness-promoting (e.g., high intensity kicks), muscular fitness-promoting (e.g., body weight activities), and sedentary relaxation activities. Thus, it was expected that differences in MVPA between the BTM and regular PE lessons would be less than those reported in previous fitness-oriented PE interventions. The 14.0 % contribution of BTM lesson MVPA to whole-day MVPA was though greater than or similar to that found in the small number of previous accelerometer studies reporting PE’s contribution to daily physical activity (e.g., [59, 60]). Collectively, these findings underscore the potential of regular PE classes to make an important contribution to health-enhancing MVPA. Further, our results suggest that the BTM intervention model made a significantly greater contribution to whole-day MVPA than regular PE lessons.

### Physical activity and sedentary time beyond BTM and PE lessons

The unadjusted between-group differences in mean MVPA for BTM vs. PE (4.2 min) and the whole-day (4.1 min) suggest that the whole-day differences were attributed to the superior MVPA during BTM compared to regular PE lessons. A 6.6 min between-group difference in school-day MVPA however, infers that outside the BTM lesson an additional 2.5 min of MVPA was accumulated over the school day. While the precise mechanisms for activity accumulation are unknown, it is plausible that individual, interpersonal, and environmental factors may have interplayed in the BTM group to stimulate additional school-day physical activity beyond the BTM lessons. The physical, social, and pedagogical environments that children interact with at school influence activity-related behaviours [61], and it has been suggested that some children may be stimulated to accumulate more physical activity when presented with active opportunities during school [61, 62]. Moreover, it is possible that the significant between-group differences in school-day and whole-day sedentary time were related to these increased activity levels. In particular, improvements in LPA and displacement of sedentary time may be important for children’s health through associations with adiposity, cardiometabolic risk, and other health outcomes [10–12]. There were though no between-group differences in physical activity and sedentary time during the after-school through evening period, which suggest that the BTM group were relatively less active by around 2.5 min in the before- or after-school periods or in the evening [63]. Because these findings are based on data from a single day we are unable to ascertain whether the BTM group truly compensated for their greater school-day MVPA by reducing their out of school activity through biologically-driven ‘activitystat’ regulation [64].

### Muscular and cardiorespiratory fitness

At T0 and T2 29 and 72 % of the BTM group, and 35 and 51 % of the COM group, respectively, achieved the FITNESSGRAM Healthy Fitness Zone criterion standard of ≥ 7 push-ups [34]. The BTM group’s improvement may have been partially influenced by the nature of the floor-based body weight-bearing movements included in the BTM lessons. The repeated administration of the push-up test protocol at T0 through T2 and resultant learning effect also likely increased test familiarisation and therefore performance among children in both groups [65]. Standing long jump performances of the children in both groups were comparable or superior to those observed in other European children [52, 66, 67]. Although a learning effect likely influenced standing long jump performance to some degree, variation in neuromuscular maturation and therefore appropriately coordinated technique possibly contributed more to the lack of observed intervention effect [52].

Distance covered during the Andersen test and estimated VO_2_ peak values were lower in both groups than reported in Danish children [37, 68], and significantly decreased between time points. Though this decrease over time was unexpected, we do not believe it reflects a true attenuation of the children’s CRF levels. The Andersen test requires children to self-pace and for some this appeared challenging over the duration of the test [69]. We observed that during the 15-s rest periods some children’s concentration waivered, and this was reflected in fluctuations in running speed during the subsequent running periods. Moreover, some children’s motivation to complete as many shuttles as possible appeared low, which was manifested in some cases by very slow running and even walking. Children’s motivation during field tests of CRF is recognised as a threat to validity [69], and it has previously been observed that children’s motivational reactions to shuttle run fitness tests are extremely variable, depending on each child’s goal profile, performance, and perceived success [70]. For these reasons the Andersen test results should be interpreted with caution.

### Enjoyment and perceived competence during BTM and PE lessons

The BTM lessons were rated as significantly more enjoyable than regular PE. This has important implications for children’s motivation to engage in physical activity as well as maintaining participation, as evidence demonstrates that experiencing enjoyment in physical activity settings, such as PE, can enhance intrinsic motivation and increase the likelihood of continued participation [71, 72]. Both groups demonstrated increased perceived competence in the T1 BTM and PE lessons. Harter’s competence motivation theory posits that successful attempts to master a skill can increase perceived competence, leading to increases in intrinsic motivation [73]. This theory has been applied in PE settings with perceived competence being shown to significantly influence children’s motivation and physical activity behaviour [74]. In addition to the independent influences of enjoyment and perceived competence on physical activity motivation, both factors influence each other in a reciprocal manner [75]. Low perceived competence in children is associated with lower levels of physical activity enjoyment [75], and enjoyment is known to increase when perceived competence is enhanced [76, 77]. This is especially true for girls [75], a point which is supported by our results showing significant improvements in girls’ perceived competence alongside increased enjoyment (albeit non-significant) during T1 BTM lessons relative to T0 PE lessons. This previous research lends credence to our findings where the BTM group increased enjoyment and perceived competence. Since these aspects of motivation are known to influence engagement and adherence to physical activity, programmes such as BTM can play a role in increasing physical activity during PE and school hours.

### Intervention fidelity

Data from the T2 survey on fidelity of lesson delivery demonstrated that the children found the BTM lessons to be challenging, motivating and adaptable to their skills levels. These favourable factors are linked to increased intervention adherence [78] as well as being predictors of physical activity [79]. Although based on limited data, results indicated that intervention fidelity was good and that intervention school teachers saw BTM as a positive influence on the children that provided motivation for physical activity engagement. Buy in and support from key stakeholders such as teachers is essential for the success of any behavioural intervention [80]. The Diffusion of Innovations model identifies stakeholders such as teachers as essential for transfer of interventions into practice through input in the decision to participate [81]. Teachers also help to provide access to the target participants, and as was the case in BTM, their continuing support is important for institutionalisation of interventions and increased likelihood of sustainability [81]. The pilot BTM intervention, while brief in duration, was highly regarded by teachers and children. Findings indicate that in future iterations of the programme, they and others may be likely to contribute to its diffusion into practice. The fact that after the study some of the teachers attended BTM instructor training to enable them to lead BTM after-school clubs is evidence of this. It should though also be noted that individual schools or school partnerships would usually be required to pay a monthly BTM licence fee. Notwithstanding the positive findings in relation to feasibility and fidelity, this cost implication may be a barrier to some schools accessing and/or sustaining the programme.

### Sample size for a cluster RCT

Sample size calculations indicated that a future RCT would need 442 children to detect a between-group difference of 6.5 min of MPA during PE. Our pilot data suggest that this increase is feasible and would help contribute to MPA during PE as well as the whole-day. Moreover, based on our pilot data, 6.5 min of MPA would equate to a 10.1 % difference between a BTM intervention and regular PE group, which is comparable to the average MVPA difference of 10.4 % reported in a recent systematic review (51) of MVPA during PE interventions. Based on our observed MPA and VPA pilot data this increase would also amount to 50 % of BTM lesson time in MVPA which reflects recommendations for health-enhancing PE [54, 55].

### Strengths and limitations

Major strengths of this pilot study were the quasi-experimental control group design used to evaluate an existing intervention programme in ‘real world’ school settings. Involvement of a lead BTM instructor ensured high quality delivery of the intervention that was made available to all Year 6 children in the intervention schools. A small number of schools participated in the study and the modest sample size may not have been powered to detect differences between groups. Further, the schools were a convenience sample selected based on their advocacy for PE and physical activity initiatives. It is possible that this may have limited the observed intervention effects, particularly in Comparison schools that strongly emphasised PE and physical activity engagement. Though it would not be the intention to exclude such schools from any future RCT, randomised sampling procedures would be applied to limit threats to internal validity. Moreover, to limit recruitment bias in a future RCT, schools would need to commit to having sufficiently flexible timetabling arrangements for them to be allocated to intervention or comparison conditions after baseline data collection. To limit the impact on BTM and PE lesson time, questionnaire and physical activity data were collected on two occasions. It is likely that there were between-lesson variations in the children’s engagement and physical activity which may not have been captured. Whole-day physical activity and sedentary time was assessed at T1 using single-day rather than multiple day accelerometer measurement protocols. The schools were reluctant for the children to take the monitors home over multiple days, so it was not possible to know whether the observed whole-day activity and sedentary levels accurately reflected the children’s typical levels of activity. The modified Andersen Test possibly compromised the accuracy of the measure of CRF. Subsequent measurement of CRF would be undertaken using alternative methods that may be less prone to variations in individual-child motivation and pacing capabilities. Intervention fidelity was investigated using a ‘light touch’ approach through the T2 child and teacher surveys. Though more in-depth process evaluation measures (e.g., lesson observations, focus groups) would have been desirable, limited staffing resources and the need to limit the study’s impact on school curriculum time prevented this.

## Conclusions

The BTM pilot programme was effective in engaging children in significantly more MPA than during regular PE. The amount of MVPA that the BTM children took part in during lessons contributed significantly more to whole-day MVPA compared to MVPA during regular PE. Moreover, enjoyment levels during the BTM lessons were significantly higher compared to regular PE. On the days when BTM lessons were scheduled, the intervention children did significantly more whole-day and school-day physical activity and less sedentary activity than COM group peers. Improved push-up and reduced CRF test performances were observed in both groups. This study has demonstrated that the BTM pilot programme was feasible to deliver in English primary schools, was enjoyed by the children, valued by the teachers, and provided favourable results indicating its potential to positively impact on physical activity, fitness, and psychosocial outcomes. On the basis of these results there is scope for this study to inform the design of a modified larger-scale cluster RCT evaluation.

## Abbreviations

ANCOVA: Analysis of covariance
ANOVA: Analysis of variance
BMI: Body mass index
BTM: Born to move intervention/group
COM: Comparison group
CRF: Cardiorespiratory fitness
IMD: Indices of Multiple Deprivation
LPA: Light intensity physical activity
MPA: Moderate intensity physical activity
MVPA: Moderate-to-vigorous intensity physical activity
PA: Physical activity
PE: Physical education
VPA: Vigorous intensity physical activity
YPAPM: Youth Physical Activity Promotion Model
